# Measured resting metabolic rate, respiratory quotient, and body composition in patients with narcolepsy: a preliminary report of a case–control study

**DOI:** 10.1038/s41598-020-67978-4

**Published:** 2020-07-03

**Authors:** Mahmoud M. A. Abulmeaty, Ahmed S. BaHammam, Ghadeer S. Aljuraiban, Ali M. Almajwal, Mona S. Aldosari

**Affiliations:** 1 Department of Community Health Sciences, Clinical Nutrition Program, King Saud University (KSU), PO Box 10219, Riyadh, 11433 Kingdom of Saudi Arabia; 2 Obesity Research and Management Unit, Medical Physiology Department, Faculty of Medicine, Zagazig University, Zagazig, Egypt; 3 University Sleep Disorders Center, College of Medicine, King Saud University (KSU), Riyadh, Kingdom of Saudi Arabia; 4 National Plan for Science and Technology, College of Medicine,, King Saud University (KSU), Riyadh, Kingdom of Saudi Arabia; 5 Department of Therapeutic Nutrition, King Khalid University Hospital, King Saud University(KSU)-Medical City, King Saud University (KSU), Riyadh, Kingdom of Saudi Arabia

**Keywords:** Fat metabolism, Endocrine system and metabolic diseases

## Abstract

This case–control study compared the body composition, resting metabolic rate (RMR), and respiratory quotient (RQ) of narcolepsy patients with those of body mass index (BMI)- gender and age-matched controls. This study included 14 male patients with narcolepsy and 14 matched controls. The narcolepsy patients were subdivided into two subgroups (n = 7/each): those with cataplexy (NT1) and those without cataplexy (NT2). Anthropometric measurements, bioelectric impedance analysis, and indirect calorimetry were used in addition to the calculation of common body-composition indices (conicity index, abdominal volume index, and body adiposity index). Our results showed no significant difference in fat percentage, fat mass, fat-free mass, and TBW among NT1, NT2, and controls (*p* > 0.05). Compared to matched controls, there was a reduction of muscle mass in both NT1 and NT2 subgroups. The RMR was similar in all groups, while patients in the NT1/NT2 subgroups had a lower RQ, used more fat and fewer carbohydrates during the fasting period. These findings give an insight into the distinctive state of altered metabolism in patients with narcolepsy, especially the resting metabolic rate, which was not altered in NT1 vs. NT2 compared to the controls when matched for BMI, age, and gender.

## Introduction

Westphal was the first to describe narcolepsy in 1877^1^. Unlike healthy individuals, people with narcolepsy enter their first rapid eye movement (REM) phase immediately after the onset of sleep^2^. Narcolepsy type 1 (NT1) is accompanied by cataplexy (sudden loss of muscle tone triggered by mostly positive emotion), whereas narcolepsy type 2 (NT2) is an absence of cataplexy^3^. Narcolepsy, particularly NT1, is associated with hypocretin (orexin-A) deficiency due to the loss of hypothalamic orexinergic neurons and leads to irresistible attacks of sleep, cataplexy, hypnagogic hallucinations, and sleep paralysis, with symptoms beginning as early as ten years of age^4,5^. There is no known cure for narcolepsy at present, although the symptoms can be managed. Several factors, such as lifestyle and genetic factors^6^, and more recently, autoimmune/inflammatory processes^7^, have been linked to the etiology of narcolepsy. The prevalence of NT1 is 25– 50 /100,000 individuals worldwide, with an incidence of 0.74/100,000 person-years^8^; the prevalence of NT2 is higher at 56/100,000 individuals^8,9^. The few available studies in Saudi Arabia have reported a prevalence of approximately 40/100,000 individuals^10,11^.

Besides sleep symptoms, several studies have documented an increase in body mass index (BMI) around the onset of narcolepsy in children^12^ and adults^13,14^. This may be attributed to the loss of orexin-A-producing neurons, which modulate feeding behavior, resting metabolic rate (RMR), respiratory quotient (RQ), and some hormones, and thus potentially exert a major influence on energy expenditure^15^. Furthermore, a study of adipose tissue distribution by MRI in patients with narcolepsy showed a distinctive pattern, including excessive subcutaneous adipose tissue rather than visceral adipose tissue and no effect on brown adipose tissue^16^. The animal models with loss of orexin develop obesity even in the presence of reduced food intake^17^. In humans, despite hypophagia, patients with narcolepsy have reported excessive weight gain^18^. However, Fronczek et al.^19^ found no differences between the resting metabolic rates (RMRs) of narcolepsy patients and those of age- and BMI-matched controls. A previous study reported obesity with a lower metabolic rate in a mouse model of narcolepsy (lacking hypocretin)^20^. However, regarding patients with NT1, research on the metabolic rate is still inconclusive^19,21^. Although the BMI increase associated with narcolepsy is well-established, the relationship between narcolepsy and other metabolic measures such as body fat/muscle distribution and RQ independent of BMI are not well documented, and the available evidence is inconsistent^15,19^. Understanding the body fat/muscle distribution of narcolepsy patients may shed a light on the mechanisms and type of weight gain that occurs in narcolepsy. Previous studies have mainly used measures such as BMI and the waist − hip ratio to measure body fat^22^, limiting the accuracy of their findings. We hypothesized that narcolepsy patients have lower RMR than that of the BMI- matched controls. Thus, this study aimed to identify the differences in the body composition, RMR, and RQ of patients with narcolepsy (type 1 and 2), and those of healthy sex- age- and BMI-matched controls using a multi-frequency bioelectric impedance analyzer to measure body composition and an indirect calorimetry device to measure RMR and RQ.

## Results

Table 1 presents the polysomnographic and multiple sleep latency (MSLT) data of patients with narcolepsy (both NT1 and NT2). Table 2 shows the self-reported demographic data. The physical activity level is significantly different among the narcolepsy subgroups and age- and BMI-matched controls. Percentage of those doing regular physical activity for 30 min in the NT1, NT2, and control groups were 0%, 0%, and 21.4%, respectively, while those who adhered to 60-min regular physical activity were 14.3%, 0%, and 7.1%, respectively. Despite being of similar age and BMI, the patients in the NT1 and NT2 were significantly taller (*F* = *7.2; df* = *2; p* = *0.003*) and had lower diastolic blood pressure (*F* = *4.8; df* = *2; p* = *0.017*). The WC, HC, and WHR were not significantly different among all groups (Table 3). The measured RMR of both the NT1 and NT2 subgroups was similar to that of the control group (*F* = *0.017; df* = *2; p* = *0.983)*. However, RQ was significantly lower in all narcolepsy subgroups (*F* = *6.3; df* = *2; p* = *0.006*). These results mean that patients in the NT1 and NT2 subgroups significantly utilized more fat (*F* = *5.8; df* = *2; p* = *0.009*) and fewer carbohydrates (*F* = *5.5; df* = *2; p* = *0.010*) in their metabolism during the fasting period. Further comparison of all parameters between the NT1 and NT2 subgroups showed no significant differences (Table 3).

**Table 1:** Polysomnographic and multiple sleep latency rest recordings of patients with narcolepsy.

Variables	Narcolepsy type 1	Narcolepsy type 2	p-value
Epworth Sleepiness scale	19.5 ± 3.5	16.9 ± 4.4	0.310
**Polysomnographic findings**			
Sleep latency (min)	5.5 ± 1.4	7.1 ± 2.2	0.222
Latency to rapid eye movement (min)	54.8 ± 12.5	96.8 ± 20.1	0.008
Sleep efficiency (%)	80.4 ± 5.7	84.5 ± 5.1	0.310
Stage N1 (%)	15.1 ± 3.3	6.9 ± 2.1	0.008
Stage N2 (%)	50.1 ± 4.2	64.8 ± 3.2	0.008
Stage N3 (%)	11.3 ± 2.2	11.5 ± 2.2	0.690
Stage R (%) (Rapid eye movement sleep)	23.5 ± 3.4	16.8 ± 2.8	0.008
Arousal index	26.2 ± 5.1	18.3 ± 4.2	0.016
**Multiple sleep latency test**			
Sleep latency (min)	2.4 ± 0.4	3.1 ± 0.6	0.032
Sleep onset rapid eye movement periods (average)	3.6 ± 0.4	2.7 ± 0.3	0.008
Rapid eye movement latency (min)	2.7 ± 0.3	4.2 ± 0.9	0.008

**Table 2:** Demographic data among study groups.

Variables	Groups			χ2	p-value
	Narcolepsy Type 1 % within subgroups	Narcolepsy Type 2 % within subgroups	Control % within subgroups		
Smoking				1.80	*0.49*
Non-smoker	57.1	57.1	85.7		
Smoker	42.9	42.9	14.3		
Physical activity				15.26	*0.01*
No regular physical activity	28.6	14.3	64.4		
Regular physical activity for 15 min	57.1	85.7	7.1		
Regular physical for 30 min	0.0	0.0	21.4		
Regular physical for 60 min	14.3	0.0	7.1		
Marital status				1.71	*0.42*
Married	28.6	57.1	57.1		
Not married	71.4	42.9	42.9		
Occupation				0.49	*0.78*
Not employee (has no fixed job)	42.9	71.4	28.6		
Employee (has a fixed job)	57.1	28.6	71.4		
Residence				2.10	*0.35*
Live outside the urban area	42.9	28.6	14.5		
Live inside the urban area	57.1	71.4	85.5

**Table 3:** Anthropometric and clinical characteristics of the study subgroups.

Variables	Narcolepsy type 1 (with cataplexy) mean ± sd (n = 7)	Narcolepsy type 2 (no cataplexy) mean ± sd (n = 7)	BMI-matched control mean ± sd (n = 14)	p-value
Age (year)	25.29 ± 3.59^a^	30.14 ± 6.15^a^	27.86 ± 4.38^a^	0.287
Height (cm)	174.86 ± 5.40^a^	172.81 ± 4.53^a^	167.82 ± 3.58^b^	0.009
Weight (kg)	102.79 ± 13.25^a^	93.19 ± 21.51^a^	92.34 ± 21.90^a^	0.212
BMI (kg/m^2^)	33.73 ± 5.18^a^	31.19 ± 7.05^a^	32.77 ± 7.54^a^	0.655
WC (cm)	110.93 ± 9.35^a^	106.86 ± 16.19^a^	101.93 ± 16.62^a^	0.221
HC (cm)	118.86 ± 8.82^a^	112.28 ± 13.55^a^	110.21 ± 15.68^a^	0.277
WHR	0.93 ± 0.07^a^	0.95 ± 0.05^a^	0.92 ± 0.05^a^	0.610
Systolic blood pressure (mmhg)	114.86 ± 11.42^a^	108.57 ± 15.12^a^	119.57 ± 9.98^a^	0.122
Diastolic blood pressure (mmhg)	75.86 ± 7.86^a^	74.14 ± 10.75^a^	85.14 ± 7.94^b^	0.017
Fasting glucose level (mg/dl)	97.43 ± 9.47^a^	100.43 ± 11.28^a^	93.43 ± 10.14^a^	0.577
Measured rmr (quark rmr)	2075.29 ± 112.57^a^	2035.14 ± 527.12^a^	2053.86 ± 434.49^a^	0.983
Respiratory quotient	0.72 ± 0.04^a^	0.73 ± 0.06^a^	0.81 ± 0.07^b^	0.006
Volume of oxygen/min	309.14 ± 17.55^a^	301.71 ± 74.52^a^	300.00 ± 64.26^a^	0.946
Volume of carbon dioxide/min	222.71 ± 15.32^a^	223.00 ± 68.67^a^	241.71 ± 52.72^a^	0.629
Percentage of utilized fat	80.11 ± 3.83^a^	73.17 ± 11.48^a^	54.71 ± 22.86^b^	0.003
Percentage of utilized carbohydrates	3.69 ± 3.38^a^	9.24 ± 13.99^a^	27.95 ± 22.05^b^	0.006

Values with the different superscripts within a raw are statistically significant according to the Bonferroni posthoc test (p < 0.05).

As shown in Table 4, differences in body composition (BFP, FM, FMI, FFM, and FFMI) among groups were not significant. However, the muscle masses of both the NT1 and NT2 subgroups were significantly lower than of the control group (34.26 ± 2.46 kg, and 31.41 ± 4.74 vs. 61.53 ± 8.60 kg, respectively; *F* = *63.9; df* = *2; p* = *0.000*), with no significant difference between the NT1 and NT2 subgroups. Compared to the control group, the TBW was insignificantly different in the NT1 and NT2 subgroups. Similarly, the CI, AVI, and BAI were not significantly differed among the study groups.

**Table 4:** Body composition parameters among study subgroups.

Variables	Narcolepsy type 1 (with cataplexy) mean ± SD (n = 7)	Narcolepsy type 2 (no cataplexy) mean ± SD (n = 7)	BMI-matched control mean ± SD (n = 14)	p-value
Body fat percent (%)	30.74 ± 5.36^a^	30.21 ± 5.21^a^	28.31 ± 8.08^a^	0.553
Fat mass (kg)	31.76 ± 10.04^a^	28.94 ± 12.21^a^	27.44 ± 14.18^a^	0.346
Fat mass index	10.48 ± 3.62^a^	9.70 ± 4.07^a^	9.74 ± 4.97^a^	0.665
Fat free mass (kg)	69.70 ± 5.22^a^	64.00 ± 9.92^a^	64.53 ± 9.02^a^	0.367
Fat free mass index	22.82 ± 1.83^a^	21.42 ± 3.13^a^	22.90 ± 2.97^a^	0.500
Muscle mass (kg)	34.26 ± 2.46^a^	31.41 ± 4.74^a^	61.53 ± 8.60^b^	0.000
Total body water (l)	51.03 ± 3.81^a^	46.87 ± 7.28^a^	53.32 ± 5.40^a^	0.104
Conicity index	1.33 ± 0.07^a^	1.34 ± 0.06^a^	1.26 ± 0.08^a^	0.095
Abdominal volume index	24.84 ± 4.26^a^	23.33 ± 7.40^a^	21.36 ± 7.04^a^	0.222
Body adiposity index	33.53 ± 5.05^a^	31.43 ± 5.81^a^	32.69 ± 7.02^a^	0.063

Values with the different superscripts within a raw are statistically significant according to the Bonferroni posthoc test (p < 0.05).

The parameters comparison of Stanford Sleepiness Scale (SSS), and symptoms between the NT1 and NT2 subgroups were shown in Table 5. The SSS showed an insignificant difference between the NT-1 and NT-2 subgroups. However, patients in the NT-1 subgroup suffered from more sleeping paralysis, hypnagogic hallucination, and cataplexy.

**Table 5:** The Stanford Sleepiness Scale and symptoms in narcolepsy type 1 vs type 2 subgroups.

Variables	Narcolepsy type 1 (with cataplexy) Mean ± SD (n = 7)	Narcolepsy type 2 (No cataplexy) Mean ± SD (n = 7)	p-value
Stanford sleepiness scale (SSS)	3.57 ± 1.81	3.57 ± 1.13	*0.248*
Suffering from interrupted sleep; n(%)	4 (57.1)	1 (14.3)	*0.094*^***†***^
Suffering from sleep paralysis; n(%)	5 (71.4)	1 (14.3)	*0.031*^***†***^
Suffering from hypnagogic hallucination; n(%)	7 (100.0)	4 (57.1)	*0.051*^***†***^
Suffering from cataplexy; n(%)	7 (100.0)	0 (0.0)	< *0.001*^***†***^

^***†***^Significance based on Chi-Square test.

In the NT2 subgroup, RMR was significantly correlated with some parameters, including Wt, BMI, WC, HC, CI, AVI, PBF, FFM, FFMI, FM, FMI, and TBW (Table 6). While, in the NT1 subgroup, the only significant correlation with RMR was Wt, PBF, and FM. The correlations of RMR in the matched-control group is very similar to the NT2 subgroup.

**Table 6:** Correlation of the measured RMR with the main study parameters in narcolepsy type 1, type 2, and control subgroups.

Variables	Narcolepsy type 1 (with cataplexy) (n = 7)	Narcolepsy type 2 (No cataplexy) (n. = 7)	BMI-matched Control group (n = 14)
**Age_(years)**			
r	0.180	− 0.334	− 0.122
Sig. (2-tailed)	0.699	0.465	0.679
**Weight (kg)**			
r	0.857*	0.964**	0.802**
Sig. (2-tailed)	0.014	0.000	0.001
**Height (cm)**			
r	0.243	0.396	0.304
Sig. (2-tailed)	0.599	0.379	0.290
**BMI (kg/m**^**2**^**)**			
r	0.536	0.786*	0.736**
Sig. (2-tailed)	0.215	0.036	0.003
**Waist circumference (cm)**			
r	0.536	0.786*	0.648*
Sig. (2-tailed)	0.215	0.036	0.012
**Hip circumference (cm)**			
r	0.739	0.607	0.863**
Sig. (2-tailed)	0.058	0.148	0.000
**Waist hip ratio**			
r	0.000	0.321	0.031
Sig. (2-tailed)	1.000	0.482	0.916
**Conicity index**			
r	− 0.111	0.655	0.481
Sig. (2-tailed)	0.812	0.111	0.081
**Abdominal volume index**			
r	0.536	0.786*	0.648*
Sig. (2-tailed)	0.215	0.036	0.012
**Body adiposity index**			
r	0.321	0.714	0.781**
Sig. (2-tailed)	0.482	0.071	0.001
**Percent body fat**			
r	0.775*	0.893**	0.398
Sig. (2-tailed)	0.041	0.007	0.159
**Fat mass (kg)**			
r	0.821*	0.893*	0.552*
Sig. (2-tailed)	0.023	0.007	0.041
**Fat mass index**			
r	0.714	0.847*	0.503
Sig. (2-tailed)	0.071	0.016	0.067
**Fat free mass (kg)**			
r	0.667	0.821*	0.793**
Sig. (2-tailed)	0.102	0.023	0.001
**Fat free mass index**			
r	0.321	0.750*	0.736**
Sig. (2-tailed)	0.482	0.050	0.003
**Muscle mass_(kg)**			
r	0.536	0.679	0.793**
Sig. (2-tailed)	0.215	0.094	0.001
**Total body water (l)**			
r	0.667	0.821*	− 0.095
Sig. (2-tailed)	0.102	0.023	0.748

**Correlation is significant at the 0.01 level (2-tailed).

*Correlation is significant at the 0.05 level (2-tailed).

## Discussion

This case–control study is one of the few studies on body composition, RMR, and RQ of narcolepsy patients. We found that the patients in the narcolepsy subgroups had a lower RQ, and lower muscle mass compared to the control group; they also utilized more fat and fewer carbohydrates, which is consistent with our hypothesis that narcolepsy patients have lower RMR compared to their BMI- matched controls. However, our findings showed that RMR and body composition measures (BFP, FM, FMI, FFM, FFMI, and TBW) were similar between the narcolepsy patients and controls.

The results of this study also revealed a similar body composition measures (except muscle mass) between the narcolepsy patients and controls, in contrast to the results of a previous case–control study on metabolic alterations in narcolepsy patients and idiopathic hypersomnia patients. The study by Poli et al., which involved the use of the BMI and waist-hip ratio to measure body fat, reported significant differences in the metabolic parameters, including WC, high-density lipoproteins, glucose/insulin ratio, and daily energy intake, between the narcolepsy patients and controls^22^. The difference in the results may be because the study populations of both studies had different ages and BMIs. The average age of the narcolepsy patients in this study was 27.2 years, whereas the mean age of the narcolepsy patients in the Poli et al. study was 38.2 years. Increasing age is associated with a redistribution of fat and lean mass because of the intra-abdominal fat accumulates more than the total fat, whereas reduced lean body mass occurs mainly due to sarcopenia^23^.

The results of this study, however, are compatible with those of a previous cross-sectional study that reported no significant group differences in the supraclavicular brown adipose tissue fat of adolescent narcolepsy patients compared to healthy participants, suggesting that the brown adipose tissue is not affected by orexin under thermoneutral conditions^16^. It is worth noting that in this study, we used a validated, multi-frequency bioelectric impedance analyzer to measure the body composition.

Additionally, though it is hypothesized that narcolepsy patients have a lower RMR due to orexin-A deficiency^18^, the exact value of RMR in narcolepsy patients is unclear; the results of one study support this hypothesis^24^, whereas another study does not^19^. These inconsistent findings may be due to the differences in the methodologies of these studies, such as varying sample sizes, unmatched age or BMI range, and absence of a standardized indirect calorimetry measurement protocol with different variability of volumes of respiratory gases. In this study, we addressed these limitations by including sex-age-and-BMI-matched controls, using a rigorous indirect calorimetry measurement protocol, and reaching the steady-state with limited variability of VO2 and VCO2 to be < 10%. Our findings do not support the hypothesis that narcolepsy patients have a lower RMR, and we found no significant case–control difference in measured RMR. Furthermore, there was no significant difference in RMR of the NT1 and NT2 subgroups. This result is in contrast to the study by Chabas et al., in which the RMR of the narcolepsy patients was reduced but not significantly, compared to the controls^24^. However, although the control group had a lower BMI, Chabas et al. did not match cases and controls for BMI^24^.

This study also found a lower RQ in narcolepsy patients (*p* < 0.05). Since RQ is considered an index of substrate oxidation (increased values indicate higher carbohydrate *vs.* fat oxidation), an elevated RQ could predict fat accumulation in the future^25^. This study showed that in the fasting state, the metabolic system of the narcolepsy subgroups consumes more fat and less carbohydrates than that of the controls during equivalent periods of fasting [RQ was in the range of fat utilization, and the percentage of utilized fat was significantly higher (Table 3, *F* = *6.3; df* = *2; p* = *0.006*)]. This higher percentage of fat oxidation and a lower percentage of carbohydrate utilization may suggest a state of insulin resistance, which is a characteristic of the metabolic system of narcolepsy patients, even at an early age^25^. Animal models with hypocretin-deficiency may develop an inability to differentiate preadipocytes in the brown adipose tissue, resulting in a reduction of thermogenesis and energy expenditure^26^. Moreover, hypocretin has been shown to regulate the metabolism of muscle glucose^27^. Collectively, these mechanisms may explain narcolepsy-associated obesity despite reduced caloric intake^14^. The insignificantly higher CI (*F* = *3.0; df* = *2; p* = *0.068*), as a measure of abdominal obesity, in narcolepsy patients supports our conclusion and may explain the possible higher frequencies of medical comorbidities such as obesity, type 2 diabetes, cardiovascular diseases, etc., in the narcolepsy population^14^. Previous studies on RQ variation in the narcolepsy patients showed that poor sleep efficiency is associated with a higher fasting RQ^29^. However, the results of other studies do not support these findings^30,31^. These inconsistencies may be due to differences in the study designs and methods of RQ measurement.

The significant correlation of RMR with anthropometric parameters such as the BMI was maintained in the control group and NT2 subgroup, while in NT1, it became insignificant. This was in line with the results of Dahmen et al.^32^ in which narcoleptic patients with lower BMI had reduced RMR. However, Dahmen et al. did not compare the NT1 vs. NT2 subgroups of the disease. Loss of the association between RMR and BMI in the NT1 subgroups might support the autoimmune or neurodegenerative etiologies of the NT1.

The major strength of this study is that it is one of the few studies that compare the body composition, RMR, and RQ of narcolepsy patients with those of gender-age-and-BMI matched controls. Previous investigations have mainly focused on the differences in the BMI of narcolepsy patients and healthy individuals^14,33^; few studies have investigated the metabolic characteristics of narcolepsy patients independent of BMI. The present study involved the use of standardized validated instruments and scales for the assessment of several parameters, which further strengthens the study. A limitation of this study is the relatively small sample size, although we calculated the needed sample size to detect the changes in RQ in the current data, the estimated sample size was 16 (see Supplementary file: Appendix 1). Nevertheless, we cannot exclude type I error, which may have decreased the power of the study. Moreover, this study did not include females or adjust for dietary intake and exercise activity. The current study assessed the RMR; therefore, future studies should determine metabolic rate during activity, because the alterations in narcolepsy might be in the active phase.

In conclusion, compared to sex-age-BMI matched control, this study showed that narcolepsy patients have a lower RQ, and muscle mass. We also found that RMR and body composition measures (BFP, FM, FMI, FFM, and FFMI) were similar between the narcolepsy patients and controls. These characteristics observed in the NT1 and NT2 patients are important for understanding the pathophysiology of the disease. Narcolepsy patients could benefit from muscle-building exercises to improve muscle mass and to lower the conicity index. Further studies of narcolepsy patients of both sexes are needed to broaden the understanding of the pathophysiology of the disease, especially regarding the metabolism of substrates.

## Subjects and methods

### Participants and recruitment

This case–control study included 14 male patients diagnosed with narcolepsy and 14 BMI- and age-matched healthy male subjects. All patients were recruited from the University Sleep Disorders Center, College of Medicine, Riyadh, Saudi Arabia, and the healthy controls were recruited from the Therapeutic Nutrition Clinic of the College of Applied Medical Sciences, King Saud University Medical City (KSUMC), King Saud University. Inclusion criteria for the patients were as follows: adult patients (> 18 years) diagnosed with narcolepsy using the International Classification of Sleep Disorders, 3rd edition (ICSD-3)^3^ and absence of comorbidities such as obstructive sleep apnea (OSA) (via polysomnography), diabetes, endocrine disorders, debilitating diseases, and psychiatric problems. The narcolepsy patients were further divided into NT1 and NT2 subgroups (n = 7, each subgroup).

Narcolepsy was diagnosed according to the International Classification of Sleep Disorders, third edition (ICSD-3)^3^. Overnight polysomnographic study (PSG) followed by a multiple sleep latency test (MSLT) was done^3^. For NT1 diagnosis, the presence of irresistible attacks of sleep, in addition to a mean sleep latency of < 8 min on the MSLT with evidence of two sleep-onset rapid eye movement periods (SOREMPs) (or one SOREMP on PSG and one or more on MSLT) and clear cataplexy “more than one episode of generally brief (< 2 min), usually bilaterally symmetrical, sudden loss of muscle tone with retained consciousness”^3^. On the other hand, NT-2 was diagnosed if the mean sleep latency was < 8 min on the MSLT and two SOREMPs (or one SOREMP on PSG and one or more on MSLT), but cataplexy was absent^3^. Other causes of excessive daytime sleepiness were excluded. Moreover, all participants were asked to maintain a minimum of 8 h in bed for 3 consecutive days before commencing the study (verified via sleep diaries).

Regarding antinarcoleptic medications, all patients in the NT1 subgroup were on modafinil therapy, while in the NT2, only 5 out of 7 were taking Modafinil. The control group consisted of 14 healthy sex-age-and-BMI-matched adult volunteers. All volunteers were interviewed by a sleep medicine specialist to rule out coexisting sleep disorders. Additionally, a validated Arabic version of the STOP-Bang questionnaire was used to assess the risk of OSA^34^**.** Those with high risk for OSA were excluded.

All participants were evaluated from December 2017 to June 2018. The Ethics Committee of the King Khalid University Hospitals, KSUMC, approved this work under the reference number IRB# E-15–1,484. Accordingly, all methods and protocols were carried out following relevant guidelines and regulations.

### Procedures

All participants attended an information session about the study, signed a written informed consent form, and filled out a health history questionnaire. All anthropometric measurements were recorded, body composition was identified using the bioelectrical impedance analysis (BIA) technique, and RMR measurement by indirect calorimetry was performed for all study participants.

### Measures

#### Self-report measures

Patient characteristics, smoking history, and physical activity history were assessed via a self-report questionnaire that each study participant filled under the supervision of a research team member. In addition to completing the SSS, a 7- statements ranging from 1 (alert) to 7 (sleep onset soon)^35^**,** and presence or absence of sleep symptoms such as interrupted sleep, sleep paralysis, hypnagogic hallucination, and cataplexy.

#### Anthropometry

Height (Ht) was measured while each participant stood with their heels, buttocks, shoulders, and occiputs touching the vertical stadiometer (Seca Model 206 stadiometer; Seca Co, Germany), and eyes looking along the Frankfort plane. Weight (Wt) was determined to 0.1 kg accuracy on a Seca scale, participants stood barefoot and wore minimal clothes. BMI was calculated with the equation Wt_(kg)_/Ht_(m)_^2^. Waist (WC) and hip (HC) circumferences were measured using inelastic tape placed at the upper edge of the iliac crest and the most prominent point of the gluteal region. The average of the two measurements was used in the analysis. The waist − hip ratio (WHR) was calculated as WC/HC^36^.

#### Body composition

A multi-frequency bioelectric impedance analyzer (TANITA BC-418 analyzer; Tanita Co, Japan) was used to measure the body fat percentage (BFP), fat mass (FM), fat-free mass (FFM), muscle mass, and total body water (TBW). The calculation of fat mass index (FMI) and fat-free mass index (FFMI), were found using the following formula: FMI = FM_(kg)_ /Ht^2^_(m_^2^_)_ and FFMI = FFM_(kg)_ /Ht^2^_(m_^2^_)_^37^. Other clinically valid anthropometric body-compositional indices were calculated using these equations: (1) The conicity index (CI), where (CI = WC_(m)_ / [0.109 × √{weight _(kg)_/height_(m)_}]^38^; (2) The abdominal volume index (AVI), where AVI = [2 × (WC)^2^ + 0.7 × (waist − hip)^2^] /1,000^39^, and (3) The body adiposity index (HC/Ht^1.5^)^40^.

#### RMR measurement

An indirect calorimetry device (QUARK RMR; COSMED, Inc., Italy) was used to measure RMR and other indirect calorimetry-related parameters of all participants. Room temperature was maintained at a comfortable level (about 25 °C). All participants were instructed to come to the indirect calorimetry lab between 8 and 11 am after fasting for at least 12 h and abstaining from strenuous physical activity and not smoking for 24 h. Every day, the device was warmed-up and calibrated before its first use. The participant was asked to lay comfortably and completely still on the bed for 16 min without sleeping or moving. Measurement sessions with at least 5 min of minor gas volume variations (VO_2_ and VCO_2_ of less than 10%) were considered indications that the participant had reached the steady-state and was used for analysis^41^. The parameters that were measured and used for analysis were RMR, O_2_ volume (VO_2_), CO_2_ volume (VCO_2_), the RQ, percentage of utilized fat (Fat%), and percentage of utilized carbohydrates (CHO%).

#### Other measures

Other clinical parameters, including systolic/diastolic blood pressure and fasting blood glucose level, were also recorded. Blood pressure was measured on the left arm of the patient using a digital sphygmomanometer (Omron Healthcare Co, Japan) while the patient sat with relaxed legs. Glucose level was checked with a digital glucometer (ACCU-CHEK, Hoffmann-La Roche Ltd, USA)^42^.

### Statistical analysis

Statistical Package for the Social Sciences (SPSS, version 25; SPSS Inc., USA) was used to analyze all data. Continuous variables were expressed as means ± standard deviations, and dichotomous variables were expressed as percentages and categories. For continuous variables, normal distribution was tested using Shapiro–Wilk test and found that RMR, RQ, VO2, VCO2, FFM, FFMI, and TBW were normally distributed (p > 0.05), while the remaining were not normally distributed. For normally distributed data, the one-way ANOVA test was used, while for variables that failed the normality test, the Kruskal–Wallis test was used to compare the means of the NT1, NT2, and control groups. Furthermore, a general linear model with the Bonferroni correction for pairwise comparison was used. The Mann–Whitney U test was used to compare means of variables between the NT1 and NT2 subgroups. Cross-tabulation and the chi-square test were used to analyze categorical variables. Spearman's correlation coefficient was used to identify the correlation between RMR and the study variables in the study subgroups.

## Electronic supplementary material

Supplementary file1 (DOCX 13 kb)

## Data Availability

The datasets generated and/or analyzed during the current study are available from the corresponding author upon reasonable request.
