# Supplementary material for: Measured resting metabolic rate, respiratory quotient, and body composition in patients with narcolepsy: a preliminary report of a case–control study
Source: Sci Rep. 2020 Jul 3;10:11024. doi: 10.1038/s41598-020-67978-4 (PMC7335078; doi:10.1038/s41598-020-67978-4)
Supplement: Supplementary file 1 — Supplementary file1 (DOCX 13 kb) [file 41598_2020_67978_MOESM1_ESM.docx]

| **Confidence Level (Z-score) at 95%:** | 1.96 |
| --- | --- |
| **Standard of Deviation:** | 0.07 |
| **Margin of Error (Confidence Interval):** | 0.05 |
|  |  |
| **Sample Size=** | **8** |
| **Sample Size (Contingency)=** | **8** |
| **Sample Size (Design Effect)(Cluster)=** | **16** |

**Sample size calculation**

n=Z-score² x StdDev²/ (margin of error)²

***Description:***

**n**= required sample size
**Z-score =**confidence level at 95% (1.96) (type I error of 5%)

**StdDev =**standard deviation of the quantitative output (from earlier published papers or pilot study)
**m =**margin of error (used value is 5%)
